# Chromatin Landscaping At Mitotic Exit Orchestrates Genome Function

**DOI:** 10.3389/fgene.2020.00103

**Published:** 2020-02-25

**Authors:** Muhammad Shoaib, Nidhi Nair, Claus Storgaard Sørensen

**Affiliations:** Biotech Research and Innovation Centre (BRIC), Faculty of Medical and Health Sciences, University of Copenhagen, Copenhagen, Denmark

**Keywords:** chromatin compaction, mitosis, decondensation, cell cycle, transcription, replication

## Abstract

Chromatin architecture is highly dynamic during different phases of cell cycle to accommodate DNA-based processes. This is particularly obvious during mitotic exit, where highly condensed rod-like chromatids need to be rapidly decondensed. Such chromatin structural transitions are tightly controlled and organized as any perturbance in this dynamic process can lead to genome dysfunction which may culminate in loss of cellular fitness. However, the mechanisms underlying cell cycle-dependent chromatin structural changes are not fully understood. In this mini review, we highlight our current knowledge of chromatin structural organization, focusing on mitotic exit. In this regard, we examine how nuclear processes are orchestrated during chromatin unfolding and compartmentalization and discuss the critical importance of cell cycle-controlled chromatin landscaping in maintaining genome integrity.

## Introduction

The cell cycle of proliferating cells is defined by two major events, first, error-free duplication of the genome during synthesis phase and second, faithful transmission of genetic material into the daughter cells during mitosis. Since genetic material is packaged in the form of chromatin, the proper execution of nuclear processes is critically dependent on cell cycle regulated chromatin organization and restructuring (Ma et al., 2015). This process is orchestrated by a variety of factors notably histone PTMs (posttranslational modifications) and chromatin protein complexes (Antonin and Neumann, 2016). In this mini review, we highlight how daughter cells inherit proper chromatin structure and discuss its importance in the execution of genome-wide nuclear functions.

## Chromatin Structure in Interphase

The fundamental repeating unit of chromatin is the nucleosome, which is formed by ~147 bp of DNA wrapped around an octamer histone core (Luger et al., 1997). Individual nucleosomes are connected by linker DNA and organized into long linear arrays, which interact with nucleosomes in the neighboring arrays to create a chromatin fiber (Luger et al., 2012; Belmont, 2014; Hansen et al., 2018). Interactions among adjacent chromatin fibers may contribute to increased folding, finally reaching the maximal degree of compaction (∼10,000 fold) observed in the metaphase chromosome (Tremethick, 2007; Batty and Gerlich, 2019). It is now widely established that chromosome territories/domains are positioned in a non-random manner within the nucleus where gene density, chromosome size and morphology play a major determining role in their organization (Nagano et al., 2017; Nozaki et al., 2017; Finn et al., 2019; Mirny et al., 2019). Gene-rich areas tend to locate at the center of the nucleus whereas gene-poor regions tend to be located at the periphery where they are associated with the nuclear lamina (NL) (Nunez et al., 2009; Van Steensel and Belmont, 2017; Lochs et al., 2019; Sivakumar et al., 2019).

**Figure 1:**
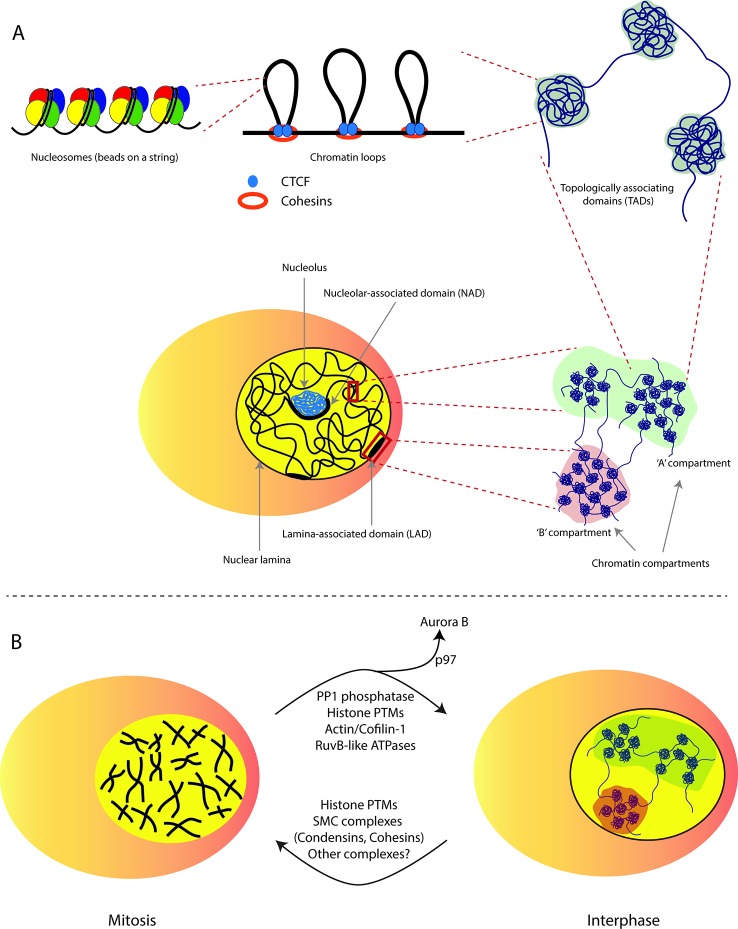
Chromatin structural organization during interphase and mitosis. **(A)** Levels of chromatin structural organization. At the most basic level, an histone octamer makes a nucleosome with ~147 bp of DNA resembling a beads-on-a-string structure, which is then folded with neighbouring nucleosomes to make a chromatin fiber. Individual fibers help establish structural chromatin loops through co-anchorage with CTCF and cohesion by means of loop extrusion. Self-interacting chromatin loops then assemble together into TADs (topologically associating domains). Several TADs then spatially organize to become specific nuclear compartments i.e., the A-type (active) enriched in active genes or the B-type (inactive) compartments that mainly comprise of repressed genomic regions including but not limited to LADs (lamina-associated domains) and NADs (nucleolar associated domains). **(B)** Chromatin structure transition from mitosis to G1 phase of the cell cycle. At the onset of mitosis, interphase chromatin is organized into highly condensed rod-shaped chromatids organized by SMC complexes (condensins and cohesins) and several phosphorylation events on histone H3. At the mitotic exit chromosomes rapidly decondense into more loosely packed, non-random interphase chromatin structures. The phosphatase PP1 plays a major role in dephosphorylation of H3S10 residue and this is deemed critical for decondensation. Nuclear targeting of actin filaments by Cofilin-1 also facilitates nuclear volume expansion presumably through structural reorganisation of the nuclear compartment. Additionally, RuvB-like ATPases are required for chromatin decondensation through as yet unknown mechanism. Finally, eviction of Aurora B kinase by the p97 ATPase is essential for chromosome decompaction as cells enter the next cell cycle.

High-throughput sequencing based approaches have markedly advanced the understanding of chromatin folding patterns and their relevance to nuclear functions. In particular, different versions of chromosome conformation capture-based methods (Denker and De Laat, 2016; Sati and Cavalli, 2017; Han et al., 2018) were developed to measure the frequency at which two genomic loci physically associate in 3D space (Sati and Cavalli, 2017). The most recent of such methods termed Hi-C measures frequencies of all the possible genomic contacts (all-versus-all). This method has been used to identify three primary landscapes of chromatin folding: i) loops, ii) TADs (topologically associating domains) and, iii) compartments (Denker and De Laat, 2016; Beagrie and Pombo, 2017; Nagano et al., 2017). Chromatin loops are formed when two small genomic regions typically 100 to 750 kb (kilobases) apart come in close proximation through association with CTCF (the CCCTC-binding factor) (Rao et al., 2014). Hi-C mapping at a higher resolution has annotated ~2,000 sharply defined regions as TADs (Dixon et al., 2012). TADs are relatively isolated genomic regions around 100 kb to 2 Mb (megabases) in size that exhibit preferential intra-domain contacts. Finally, at the Mb scale, chromosomes are segregated into A-type (active) and B-type (inactive) compartments that are defined by their transcriptional activity (Simonis et al., 2006) (**Figure 1A**). In the context of this review, we focus on the major components of the B-type compartment, including LADs (lamina-associated domains), NADs (nucleolar-associated domains), as described in **Box 1**.

Box 1 **Lamina-associated domains (LADs)**

Condensed chromatin regions corresponding to B-type domains that lie in proximity to the nuclear lamina (NL) are termed as lamina-associated domains (LADs) (Van Steensel and Belmont, 2017). There are approximately 1,000–1,500 LADs, typically 0.1–10 Mb in size that cover more than one-third of the genome and are distributed along all chromosomes (Guelen et al., 2008). LADs have sharply defined borders enriched for active promoters leading away from the LADs, CpG islands and CTCF binding sites.

**Nucleolar-associated domains (NADs)**

Nucleolar-associated domains (NADs) are heterochromatic regions that associate with the nucleolus (Nemeth et al., 2010; Van Koningsbruggen et al., 2010). NADs are relatively gene-poor, enriched in satellite repeats and approximately 0.1–10 Mb in size. NADs are formed by active processes through tethering proteins in addition to mere physical proximity to the nucleolus (Potapova and Gerton, 2019). There is substantial overlap between NADs and LADs with some studies showing that these loci could switch positions following mitotic exit (Kind et al., 2013; Ragoczy et al., 2014). Additionally, NADs are also found to locate near to the NL in a subset of cells.

## Chromatin Structure Dynamics Around Mitosis

### Chromatin Condensation During Mitosis

The massive structural reorganization of chromatin during mitosis is mediated by the eukaryotic members of SMC (structural-maintenance-of-chromosomes) protein complexes, namely condensins and cohesins (Belmont, 2006; Wood et al., 2010; Houlard et al., 2015; Piskadlo and Oliveira, 2017). Together with topoisomerase II and other non-histone proteins, condensins and cohesins help orchestrate higher order chromatin folding and chromosome-wide compaction leading to cytologically distinct and longitudinally compacted chromosomes (Hirano, 2012; Antonin and Neumann, 2016; Piskadlo and Oliveira, 2017; Schalbetter et al., 2017). Whereas condensins organize and condense large-scale chromosome rearrangements by loop formation and lateral/axial compaction, histone PTMs generally promote inter-nucleosomal association and hence, drive close-range chromosome compaction (Antonin and Neumann, 2016). Among the various histone PTMs, phosphorylation of several of the histone H3 amino acid residues surge during different stages of mitosis (Sawicka and Seiser, 2012; Wang and Higgins, 2013), however, the exact mechanism by which they contribute to mitotic chromosome condensation in mammalian cells remains elusive.

In addition to histone H3 phosphorylation, different methylation states of histone H4 lysine 20 (H4K20me1/2/3) have also been implicated in chromatin compaction (Houston et al., 2008; Oda et al., 2009). However, the mechanistic details of their role in chromosome condensation are not well understood. SET8, the enzyme responsible for genome-wide deposition of H4K20 monomethylation, is tightly regulated during the cell cycle and peaks around G2 phase (Tardat et al., 2010; Jorgensen et al., 2013). Majority of H4K20me1 is subsequently converted into H4K20me2 and H4K20me3 by the action of SUV4-20H1 and SUV4-20H2 enzymes during M and G1 phases (Nishioka et al., 2002). However, about 10% of H4K20me1 persists and is found to be significantly enriched in the gene bodies of highly transcribing genes (Barski et al., 2007; Van Nuland and Gozani, 2016). The presence of H420me1 in transcriptionally active and hence, more open chromatin regions suggest that chromatin compaction functions are most likely regulated by H4K20me2 and H4K20me3 states. These observations support the idea that both SMC and histone PTM-mediated chromosome structural changes may function in parallel, albeit at different levels of chromosome architecture. It is highly likely that mitotic chromosome condensation requires a cross-talk between both these mechanisms (**Figure 1B**).

### Chromatin Decondensation After Mitosis

Mitotic exit is characterized by two major nuclear events, first, the nuclear envelope is reformed to provide an enclosed space for the segregated genomic material. Second, the re-establishment of functional interphase chromatin within the nuclear envelope, where rod-shaped chromatids rapidly decondense into more loosely arranged, non-random structures, fully competent for DNA-based processes. Indeed, simulation on a mitotic chromosome-like polymer shows that the large-scale 3D organization of TADs and A/B compartments during mitotic exit occurs simply as a result of partial decondensation in an inflation-like process (Kumar et al., 2019). In this regard, while TADs and loops are established rapidly following mitotic exit, the larger A/B compartments form more slowly and continue to grow as cells advance through the cell cycle (Abramo et al., 2019).

In the context of this review, we focus on how the major B-type compartment components i.e. LADs and NADs, are organized at mitotic exit. During interphase, LADs interact dynamically with the NL however, they move only within a layer <1 µm thick (Kind et al., 2013). Furthermore, there is a degree of both cooperativity and stochasticity in the positioning of LADs within individual cells (Jurisic et al., 2018). Intriguingly, the nuclear positioning of majority of LADs is not inherited following mitosis but instead some LADs (termed facultative LADs or fLADs) are stochastically reshuffled between other repressive environments. However, around 30% of LAD regions, termed cLADs (constitutive LADs) appear to be cell-type invariant in their association with the nuclear periphery and may serve to anchor chromosomes to the NL (Kind et al., 2015). Anchoring of cLADs, that display the highest NL contact frequencies and form the most stable NL contacts, likely contributes to the overall organization of interphase chromatin after mitotic exit (Falk et al., 2019). In this regard, H3K9me2 (histone H3 lysine 9 dimethyl) has recently been shown to be preserved across mitosis and is required for the re-establishment of LADs in the daughter cells (Poleshko et al., 2019).

NADs are also found to locate in the proximity of NL in a subset of cells. In this regard, there may be a substantial overlap between NADs and LADs with some studies showing that these loci could switch positions following mitotic exit (Van Koningsbruggen et al., 2010; Kind et al., 2013; Ragoczy et al., 2014). A recent study identified two distinct classes of NADs in mouse embryonic fibroblasts, which differ primarily in their frequency to associate with the nucleolar periphery and with the NL (Vertii et al., 2019). While type I NADs display characteristics of constitutive heterochromatin and associate with both nucleolar periphery and NL, type II NADs are more specifically associated with the nucleolus. Considering a substantial overlap between type I NADs and LAD regions, their mode of inheritance is expected to be largely the same (Kind et al., 2013; Vertii et al., 2019). However, it is unclear at the moment how type II NADs are inherited in the daughter cells.

At the nucleosomal level, the chromatin landscaping at mitotic exit is marked primarily by reappearance of histone acetyl marks and loss of histone phosphorylation (Wang and Higgins, 2013). The phosphatase PP1 plays an essential role in removing mitotic H3 phosphorylation, including H3T3p, H3S10p, H3T11p, and H3S28p. In this regard, Repo-Man, the principal PP1-recruiting factor is targeted to anaphase chromosomes and is required for timely removal of H3T3p and H3T11p (Qian et al., 2011; Vagnarelli et al., 2011). Although Repo-Man is dispensable for chromatin decondensation at mitotic exit, it has been shown to play a role in nuclear envelope reformation in a PP1-independent manner (Vagnarelli et al., 2011). In this regard, the nucleolar protein Ki-67 might function redundantly with Repo-Man to target PP1 onto anaphase chromosomes (Booth et al., 2014). Further into mitosis, PNUTS, the PP1 nuclear targeting subunit localizes PP1 to the reforming nuclei and its loading onto chromatin has been linked to decondensation (Landsverk et al., 2005). However, as this occurs following dephosphorylation of H3S10, the exact mechanism by which PNUTS facilitates chromosome decompaction is not currently understood. Removal of H3S10p leads to the dissociation of the chromosome passenger complex and promotes re-establishment of HP1 (heterochromatin protein 1) binding to H3K9me3 (histone H3 lysine 9 trimethyl) to maintain heterochromatin at mitotic exit. Establishment of the H3K9me3-HP1 axis facilitates loading of cohesin by the histone H4K20 methyltransferase SUV4-20H2 that is itself targeted through HP1 binding. This initial loading of cohesin seems to be crucial for the establishment of pericentromeric heterochromatin as cells enter interphase (Hahn et al., 2013). Furthermore, H4K20 methylation is in itself important for finetuning chromatin compaction states during mitotic exit. In this context, we have uncovered that loss of H4K20me leads to abnormal chromatin decompaction in cells exiting mitosis, which has significant functional implications in terms of DNA replication and genome stability during the next cell cycle (discussed below) (Shoaib et al., 2018).

Additional protein complexes have also been shown to play a role in chromatin decondensation at the mitotic exit. Firstly, removal of the mitotic kinase Aurora B from the chromatin seems to be a prerequisite for chromatin decondensation and nuclear envelope reformation. This is carried out by the hexameric ATPase p97 that binds to the ubiquitylated form of Aurora B and evicts it from the chromatin thereby, facilitating chromatin decondensation (Ramadan et al., 2007). Apart from p97, a second class of ATPases, RuvBL1 and RuvBL2, seems to be essential for chromatin decondensation. Using purified chromatin and Xenopus egg extracts to recapitulate mitotic exit events in a cell free system, Magalska et al. showed that decompaction of metaphase chromosomes is an active process that requires the activity of these AAA-ATPases (Magalska et al., 2014). Finally, nuclear actin filament (F-actin) polymerization during early G1 phase of the cell cycle is thought to aid nuclear volume expansion and chromatin decondensation (Baarlink et al., 2017). In this context, the nuclear targeting of actin-disassembling factor Cofilin-1 during mitotic exit spatiotemporally controls the assembly and turnover of F-actin polymers in turn regulating chromatin reorganization and nuclear architecture of the newly formed daughter cells (**Figure 1B**).

Establishing proper ‘ground state’ chromatin structure entails massive structural reorganization of the chromatin. Using single-cell Hi-C analysis, Nagano et al. compared chromatin structure in different cell cycle phases, starting from mitotic exit (Nagano et al., 2017). As the cells exit mitosis, a dramatic expansion of TADs containing active genes was observed, which subsequently decreases as cells enter S phase. On the contrary, compartmentalization increases as the cells progress through the cell cycle and reaches its peak before next mitosis (Nagano et al., 2017). More recently, detailed Hi-C mapping at defined time points following mitotic exit was presented to describe the reorganization of chromatin landscape specifically at the M-G1 transition (Abramo et al., 2019; Zhang et al., 2019). Similar to Nagano et. al., the authors observed that TADs and A/B compartments establish rapidly after mitosis and continue to strengthen through the cell cycle. Local compartmentalization is accompanied by contact domain formation in a “bottom-up” manner where smaller sub-TADs are the first to form followed by their convergence into multi-domain TAD structures. Interestingly, Zhang et al. found that CTCF is strongly retained at a significant proportion of its binding sites in mitotic chromosomes, whereas, cohesin is completely evicted during mitosis and is only loaded onto chromatin with delayed kinetics. Intriguingly, cohesin binding is followed by the formation of structural chromatin loops co-anchored with CTCF. Furthermore, the authors showed that chromatin loops can also be formed through contact between cis-regulatory elements (promotor-enhancer loops). These data suggest that a dynamic hierarchical network of mutually influential, yet distinct forces drive post-mitotic chromatin landscaping.

## Chromatin Landscaping At Mitotic Exits Meets Nuclear Function

The large-scale spatial segregation of locally folded loops, TADs, compartments that define interphase 3D chromatin organization is largely absent in mitotic chromosomes (Nagano et al., 2017; Abramo et al., 2019). Thus, during mitotic exit, chromatin is not simply decondensed but also needs to be landscaped into hierarchically folded chromatin domains. Additionally, the *de novo* establishment of functional chromatin domains needs to be well coordinated with the genome-wide execution of DNA-based processes in particular transcription and replication. In this regard, it is unclear at the moment whether nuclear functions (transcription, replication etc.) drive chromatin domain unfolding or vice versa. Below we discuss how cells coordinate chromatin reorganization and nuclear processes during their transition to the next cell cycle.

### Coordinated Transcription Around Mitosis

To achieve maximum chromatin condensation during mitosis, the landscape of interphase chromatin including intra- and inter-chromosomal contacts is lost. In this regard, many chromatin modifiers and transcription factors are dissociated from chromatin, facilitating segregation of genomic material into the daughter nuclei (Kadauke and Blobel, 2013; Naumova et al., 2013; Festuccia et al., 2017; Raccaud and Suter, 2018; Zaidi et al., 2018). In contrast to previous reports that all bound proteins are evicted from chromatin during mitosis, the histone H3K4 methyltransferase MLL1 (Mixed Lineage Leukemia 1) seems to retain its chromatin association during mitosis and its loss impairs the rapid reactivation of its target genes (Blobel et al., 2009; Black et al., 2016). Thus, a comprehensive analysis of mitotic chromosome bound proteome is required to identify whether other chromatin modifying complexes similar to MLL1 remain on the mitotic chromosome and facilitate inheritance of transcriptional competence in the daughter cells.

Additionally, recent evidence indicates ongoing transcription of many genes during mitosis albeit at low levels, with a transient surge at the mitotic exit (Palozola et al., 2017). The initial transcriptional activity following mitosis primarily relates to the genes that are involved in growth and restoration of daughter cells besides establishing the transcriptional amplitude to be later maintained during interphase. Intriguingly, around 50% of active genes exhibit this transcriptional spike, which constitutes the maximum transcriptional output per DNA copy observed at any point during the cell cycle (Blobel et al., 2009; Black et al., 2016). In terms of histone modifications, mitotic levels of histone H3 lysine 27 acetylation at the individual loci best predict the transcriptional spike seen during the M-G1 transition. These observations support the idea of ‘mitotic bookmarking’, where retention of key chromatin factors during mitosis contributes to maintenance of epigenetic memory for rapid establishment of transcriptional and structural states of the genome in the daughter cells (Kadauke and Blobel, 2013; Wang and Higgins, 2013; Ma et al., 2015; Zaidi et al., 2018). It is yet to be established how these bookmarking factors drive the formation of TADs and facilitate compartmentalization into active and inactive compartments at the mitotic exit.

**Figure 2:**
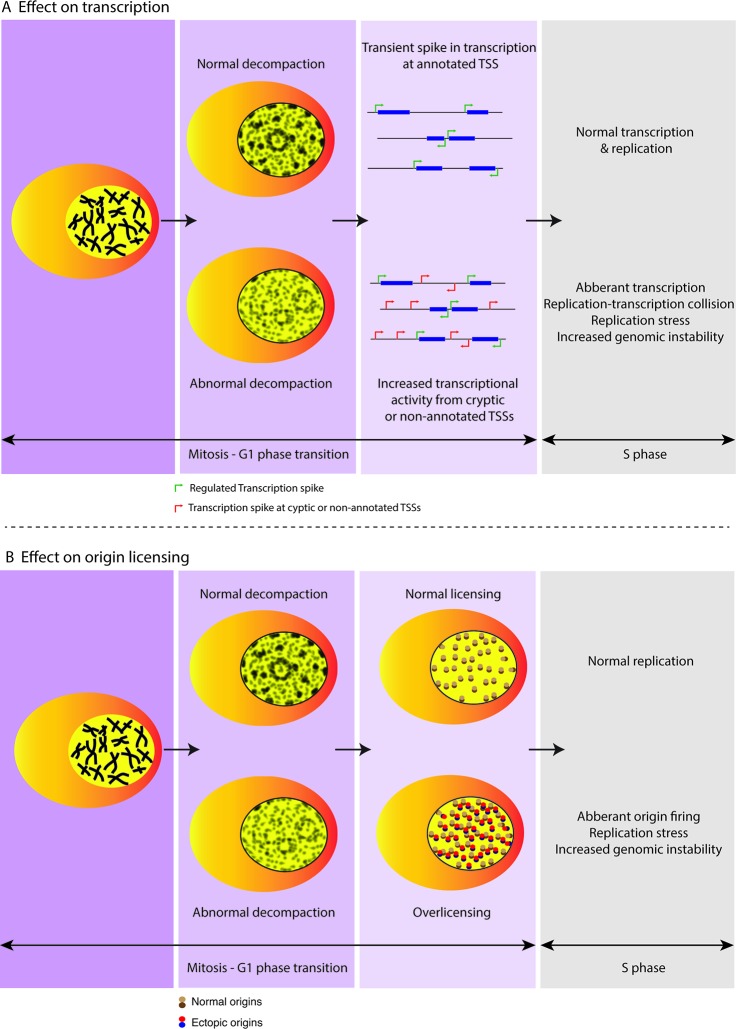
Regulated decompaction at M-G1 transition preserves genome stability. **(A)** A transient spike in transcriptional output from annotated TSSs (transcription start sites) is observed during M-G1 transition while chromatin undergoes regulated decompaction and before cells enter interphase. However, increased decompaction at this point could lead to dysregulation of gene activity wherein unplanned transcription at cryptic promoters or non-annotated TSSs could lead to replication-transcription collisions in turn causing replication stress and genomic instability further into the cell cycle. **(B)** Regulated decompaction at M-G1 transition facilitates restricted licensing of origins in preparation for DNA replication during the following S phase. However, in case of abnormal decompaction, increased chromatin accessibility is accompanied by over-licensing that can lead to replication stress and genome instability arising from aberrant origin firing at the start of subsequent S phase.

Establishing adequate chromatin compaction during G1 phase is also necessary for preventing unregulated transcription. Using DNMT (DNA methyltransferase) and HDAC (histone deacetylase) inhibitors, Brocks et al. showed that disruption of repressive chromatin environment induces cryptic transcription start sites encoded within long terminal repeat retrotransposons (Brocks et al., 2017). Recent work has further shown that condensed chromatin may not necessarily impair transcription initiation but instead leads to inefficient elongation resulting in accumulation of RNA polymerase II at transcription site (Vankova Hausnerova and Lanctot, 2017a). Upon decompaction, release of the RNA polymerase II leads to a transient increase in transcriptional activity. This transient outburst of transcription in cells undergoing mitotic exit occurs likely as a result of the rapid decondensation of chromatin before cells establish ground state chromatin (Vankova Hausnerova and Lanctot, 2017b). Hence, controlled decompaction during mitotic exit critically prevents increased &/or untoward transcriptional activity until cells have advanced further into the interphase (**Figure 2A**). This is in line with the notion that regulated chromatin decompaction during M-G1 transition is essential to ensure well-controlled DNA-based processes and thereby critical to maintainance of genomic stability (Nair et al., 2017).

### Chromatin Decondensation at Mitotic Exit Is Coupled to DNA Replication Licensing

DNA replication is a tightly controlled chromatin process that ensures faithful duplication of genetic material once per cell cycle. Replication is temporally divided into two-steps, where first the future replication origins are ‘licensed’ by loading of pre-RC (pre-replication complex) starting in late telophase and continued through G1 phase, followed by ‘firing’ of origins at the start of S phase (Remus and Diffley, 2009; Fragkos et al., 2015; Yeeles et al., 2015). Pre-RC assembly starts with loading of ORC1-6 (origin recognition complex subunits 1-6), whose binding in higher eukaryotes is largely sequence independent (Mechali et al., 2013). Later, CDC6 (Cell Division Cycle 6) and CDT1 (Chromatin Licensing and DNA Replication Factor 1) act to recruit the replicative helicase MCM2-7 (minichromosome maintenance protein complex 2-7) to ORC-bound genomic loci. Since the assembly of ORC complex starts in late mitosis, it needs to be coupled with chromosome decondensation and chromatin reorganization into functional territories and domains. The exact mechanism of recruitment of ORC complex to chromatin is not yet elucidated, however, it has been shown that ORC1, the largest subunit of ORC complex, is the first subunit to bind to mitotic chromosomes at the start of mitosis, followed by the rest of the subunits in late mitosis (Kara et al., 2015). The absence of DNA sequence specific binding of ORC complex allows a more chromatin-regulated recruitment and loading process (Cayrou et al., 2015). In this regard, the N-terminal BAH domain of ORC1 has been shown to specifically recognize H4K20me2, which in itself is being established on histone H4 around late mitosis and early G1 phase (Kuo et al., 2012). ORC complex has also been shown to interact with three repressive chromatin marks namely, H3K9me3, H3K27me3 and H4K20me3 (Bartke et al., 2010; Vermeulen et al., 2010). These findings strongly argue for a key role of specific chromatin environment that stabilizes ORC1 at human replication origins during mitosis and early G1 phase. However, it is not clear at the moment if ORC complex loading and eventually pre-RC loading is dictated or affected by chromatin loops and TADs in the daughter nuclei.

Chromatin enforces specificity of replication initiation by restricting non-specific ORC binding to origins (Devbhandari et al., 2017; Kurat et al., 2017). Thus, tightly regulated chromatin compaction threshold limits replication licensing at the M/G1 transition. In particular, recent data from our group showed that the H4K20me pathway plays a key role in establishment of ground-state chromatin compaction upon mitotic exit (Shoaib et al., 2018). In the absence of proper H4K20me levels, aberrant loading of ORC and MCM2-7 complexes promotes single-stranded DNA formation and DNA damage in the ensuing S phase. Importantly, restoration of chromatin compaction at the cellular transition from mitosis to G1 restricts uncontrolled replication licensing and thus preserves genome stability. In line with this, Kurat et al. previously showed that while chromatin does not completely inhibit assembly of CMG (CDC45/MCM/GINS) complex, DNA synthesis is strongly restricted due to the presence of chromatin and requires additional factors for replisome progression (Kurat et al., 2017). Additionally, “open chromatin” can induce replication stress by facilitating activation of dormant replication origins further threatening the fidelity of DNA replication (Conti et al., 2010) (**Figure 2B**). Finally, re-establishment of interphase chromatin domains is important for maintaining replication timing. TADs represent stable regulatory units of replication timing in a cell-type specific manner and follow characteristics of active and repressed compartments of the genome (Pope et al., 2014). In this regard, DNA replication is synchronized with transcription, initiating within the TADs permissive for transcription and later advance into repressive TAD regions. Aberrant chromatin reorganization at mitotic exit could effectively abolish TAD boundaries and hence, may endanger genomic integrity through replication-transcription conflicts.

## Conclusions/Perspectives

To coordinate and regulate various nuclear functions, chromatin inherited by the daughter cells during mitotic exit maintains the structural organization of their predecessor. It is an important mechanism for cells to maintain their cellular identity. This inevitably requires highly regulated chromatin decondensation, which is dictated by both chromatin modifications and non-histone chromatin structural proteins. The molecular events leading to reversal of highly condensed chromosomes into loosely organized interphase chromatin are not fully elucidated. In this regard, several key questions require further investigations. A comprehensive analysis of chromatin factors that remain on mitotic chromosomes during cell division is lacking. Also, it is unresolved at the moment how much mitotic bookmarking contributes to reestablishment of interphase chromatin states and how extensive it is throughout the genome. For certain genomic regions such as LADs, there is a *de novo* establishment of chromatin state (cLADs vs fLADs) at mitotic exit. It is unclear how cells push certain LAD regions to the nuclear periphery while others remain in the nuclear interior. The question remains if chromatin landscaping at mitotic exit is largely a stochastic process or there is a method to this randomness. By employing high throughput ‘omics’ approaches, future studies will shed light on chromatin landscaping at mitotic exit and how it regulates nuclear processes thereby maintaining genome integrity and cell identity.

## Author Contributions

MS, NN, and CS contributed to the conception, writing, and checking of the manuscript for important intellectual content.

## Funding

This work was created by MS, NN, and CS based on the support from the Benzon Foundation, The Novo Nordisk Foundation, The Danish Cancer Society, and The Danish Medical Research Council.

## Conflict of Interest

The authors declare that the research was conducted in the absence of any commercial or financial relationships that could be construed as a potential conflict of interest.

## References

[B1] Abramo K. Valton A. L. Venev S. V. Ozadam H. Fox A. N. Dekker J. (2019). A chromosome folding intermediate at the condensin-to-cohesin transition during telophase. Nat. Cell Biol. 21, 1393–1402. 10.1038/s41556-019-0406-2 31685986 PMC6858582

[B2] Antonin W. Neumann H. (2016). Chromosome condensation and decondensation during mitosis. Curr. Opin. Cell Biol. 40, 15–22. 10.1016/j.ceb.2016.01.013 26895139

[B3] Baarlink C. Plessner M. Sherrard A. Morita K. Misu S. Virant D. (2017). A transient pool of nuclear F-actin at mitotic exit controls chromatin organization. Nat. Cell Biol. 19, 1389–1399. 10.1038/ncb3641 29131140

[B4] Barski A. Cuddapah S. Cui K. Roh T. Y. Schones D. E. Wang Z. (2007). High-resolution profiling of histone methylations in the human genome. Cell 129, 823–837. 10.1016/j.cell.2007.05.009 17512414

[B5] Bartke T. Vermeulen M. Xhemalce B. Robson S. C. Mann M. Kouzarides T. (2010). Nucleosome-interacting proteins regulated by DNA and histone methylation. Cell 143, 470–484. 10.1016/j.cell.2010.10.012 21029866 PMC3640253

[B6] Batty P. Gerlich D. W. (2019). Mitotic chromosome mechanics: how cells segregate their genome. Trends Cell Biol. 29, 717–726. 10.1016/j.tcb.2019.05.007 31230958

[B7] Beagrie R. A. Pombo A. (2017). Cell cycle: continuous chromatin changes. Nature 547, 34–35. 10.1038/547034a 28682328

[B8] Belmont A. S. (2006). Mitotic chromosome structure and condensation. Curr. Opin. Cell Biol. 18, 632–638. 10.1016/j.ceb.2006.09.007 17046228

[B9] Belmont A. S. (2014). Large-scale chromatin organization: the good, the surprising, and the still perplexing. Curr. Opin. Cell Biol. 26, 69–78. 10.1016/j.ceb.2013.10.002 24529248 PMC3927141

[B10] Black K. L. Petruk S. Fenstermaker T. K. Hodgson J. W. Caplan J. L. Brock H. W. (2016). Chromatin proteins and RNA are associated with DNA during all phases of mitosis. Cell Discovery 2, 16038. 10.1038/celldisc.2016.38 27807477 PMC5078801

[B11] Blobel G. A. Kadauke S. Wang E. Lau A. W. Zuber J. Chou M. M. (2009). A reconfigured pattern of MLL occupancy within mitotic chromatin promotes rapid transcriptional reactivation following mitotic exit. Mol. Cell 36, 970–983. 10.1016/j.molcel.2009.12.001 20064463 PMC2818742

[B12] Booth D. G. Takagi M. Sanchez-Pulido L. Petfalski E. Vargiu G. Samejima K. (2014). Ki-67 is a PP1-interacting protein that organises the mitotic chromosome periphery. Elife 3, e01641. 10.7554/eLife.01641 24867636 PMC4032110

[B13] Brocks D. Schmidt C. R. Daskalakis M. Jang H. S. Shah N. M. Li D. (2017). DNMT and HDAC inhibitors induce cryptic transcription start sites encoded in long terminal repeats. Nat. Genet. 49, 1052–1060. 10.1038/ng.3889 28604729 PMC6005702

[B14] Cayrou C. Ballester B. Peiffer I. Fenouil R. Coulombe P. Andrau J. C. (2015). The chromatin environment shapes DNA replication origin organization and defines origin classes. Genome Res. 25, 1873–1885. 10.1101/gr.192799.115 26560631 PMC4665008

[B15] Conti C. Leo E. Eichler G. S. Sordet O. Martin M. M. Fan A. (2010). Inhibition of histone deacetylase in cancer cells slows down replication forks, activates dormant origins, and induces DNA damage. Cancer Res. 70, 4470–4480. 10.1158/0008-5472.CAN-09-3028 20460513 PMC2880188

[B16] Denker A. De Laat W. (2016). The second decade of 3C technologies: detailed insights into nuclear organization. Genes Dev. 30, 1357–1382. 10.1101/gad.281964.116 27340173 PMC4926860

[B17] Devbhandari S. Jiang J. Kumar C. Whitehouse I. Remus D. (2017). Chromatin constrains the initiation and elongation of DNA replication. Mol. Cell 65, 131–141. 10.1016/j.molcel.2016.10.035 27989437 PMC5256687

[B18] Dixon J. R. Selvaraj S. Yue F. Kim A. Li Y. Shen Y. (2012). Topological domains in mammalian genomes identified by analysis of chromatin interactions. Nature 485, 376–380. 10.1038/nature11082 22495300 PMC3356448

[B19] Falk M. Feodorova Y. Naumova N. Imakaev M. Lajoie B. R. Leonhardt H. (2019). Heterochromatin drives compartmentalization of inverted and conventional nuclei. Nature 570, 395–399. 10.1038/s41586-019-1275-3 31168090 PMC7206897

[B20] Festuccia N. Gonzalez I. Owens N. Navarro P. (2017). Mitotic bookmarking in development and stem cells. Development 144, 3633–3645. 10.1242/dev.146522 29042475

[B21] Finn E. H. Pegoraro G. Brandao H. B. Valton A. L. Oomen M. E. Dekker J. (2019). Extensive heterogeneity and intrinsic variation in spatial genome organization. Cell 176 1502–1515, e1510. 10.1016/j.cell.2019.01.020 30799036 PMC6408223

[B22] Fragkos M. Ganier O. Coulombe P. Mechali M. (2015). DNA replication origin activation in space and time. Nat. Rev. Mol. Cell Biol. 16, 360–374. 10.1038/nrm4002 25999062

[B23] Guelen L. Pagie L. Brasset E. Meuleman W. Faza M. B. Talhout W. (2008). Domain organization of human chromosomes revealed by mapping of nuclear lamina interactions. Nature 453, 948–951. 10.1038/nature06947 18463634

[B24] Hahn M. Dambacher S. Dulev S. Kuznetsova A. Y. Eck S. Worz S. (2013). Suv4-20h2 mediates chromatin compaction and is important for cohesin recruitment to heterochromatin. Genes Dev. 27, 859–872. 10.1101/gad.210377.112 23599346 PMC3650224

[B25] Han J. Zhang Z. Wang K. (2018). 3C and 3C-based techniques: the powerful tools for spatial genome organization deciphering. Mol. Cytogenet. 11, 21. 10.1186/s13039-018-0368-2 29541161 PMC5845197

[B26] Hansen J. C. Connolly M. Mcdonald C. J. Pan A. Pryamkova A. Ray K. (2018). The 10-nm chromatin fiber and its relationship to interphase chromosome organization. Biochem. Soc. Trans. 46, 67–76. 10.1042/BST20170101 29263138 PMC5818668

[B27] Hirano T. (2012). Condensins: universal organizers of chromosomes with diverse functions. Genes Dev. 26, 1659–1678. 10.1101/gad.194746.112 22855829 PMC3418584

[B28] Houlard M. Godwin J. Metson J. Lee J. Hirano T. Nasmyth K. (2015). Condensin confers the longitudinal rigidity of chromosomes. Nat. Cell Biol. 17, 771–781. 10.1038/ncb3167 25961503 PMC5207317

[B29] Houston S. I. Mcmanus K. J. Adams M. M. Sims J. K. Carpenter P. B. Hendzel M. J. (2008). Catalytic function of the PR-Set7 histone H4 lysine 20 monomethyltransferase is essential for mitotic entry and genomic stability. J. Biol. Chem. 283, 19478–19488. 10.1074/jbc.M710579200 18480059 PMC2443654

[B30] Jorgensen S. Schotta G. Sorensen C. S. (2013). Histone H4 lysine 20 methylation: key player in epigenetic regulation of genomic integrity. Nucleic Acids Res. 41, 2797–2806. 10.1093/nar/gkt012 23345616 PMC3597678

[B31] Jurisic A. Robin C. Tarlykov P. Siggens L. Schoell B. Jauch A. (2018). Topokaryotyping demonstrates single cell variability and stress dependent variations in nuclear envelope associated domains. Nucleic Acids Res. 46, e135. 10.1093/nar/gky818 30215776 PMC6294560

[B32] Kadauke S. Blobel G. A. (2013). Mitotic bookmarking by transcription factors. Epigenet. Chromatin 6, 6. 10.1186/1756-8935-6-6 PMC3621617 23547918

[B33] Kara N. Hossain M. Prasanth S. G. Stillman B. (2015). Orc1 binding to mitotic chromosomes precedes spatial patterning during G1 Phase and assembly of the origin recognition complex in human cells. J. Biol. Chem. 290, 12355–12369. 10.1074/jbc.M114.625012 25784553 PMC4424365

[B34] Kind J. Pagie L. Ortabozkoyun H. Boyle S. De Vries S. S. Janssen H. (2013). Single-cell dynamics of genome-nuclear lamina interactions. Cell 153, 178–192. 10.1016/j.cell.2013.02.028 23523135

[B35] Kind J. Pagie L. De Vries S. S. Nahidiazar L. Dey S. S. Bienko M. (2015). Genome-wide maps of nuclear lamina interactions in single human cells. Cell 163, 134–147. 10.1016/j.cell.2015.08.040 26365489 PMC4583798

[B36] Kumar R. Lizana L. Stenberg P. (2019). Genomic 3D compartments emerge from unfolding mitotic chromosomes. Chromosoma 128, 15–20. 10.1007/s00412-018-0684-7 30357462 PMC6394678

[B37] Kuo A. J. Song J. Cheung P. Ishibe-Murakami S. Yamazoe S. Chen J. K. (2012). The BAH domain of ORC1 links H4K20me2 to DNA replication licensing and Meier-Gorlin syndrome. Nature 484, 115–119. 10.1038/nature10956 22398447 PMC3321094

[B38] Kurat C. F. Yeeles J. T. P. Patel H. Early A. Diffley J. F. X. (2017). Chromatin controls DNA replication origin selection, lagging-strand synthesis, and replication fork rates. Mol. Cell 65, 117–130. 10.1016/j.molcel.2016.11.016 27989438 PMC5222724

[B39] Landsverk H. B. Kirkhus M. Bollen M. Kuntziger T. Collas P. (2005). PNUTS enhances *in vitro* chromosome decondensation in a PP1-dependent manner. Biochem. J. 390, 709–717. 10.1042/BJ20050678 15907195 PMC1199665

[B40] Lochs S. J. A. Kefalopoulou S. Kind J. (2019). Lamina associated domains and gene regulation in development and cancer. Cells 8 (3), E271. 10.3390/cells8030271 30901978 PMC6468596

[B41] Luger K. Mader A. W. Richmond R. K. Sargent D. F. Richmond T. J. (1997). Crystal structure of the nucleosome core particle at 2.8 A resolution. Nature 389, 251–260. 10.1038/38444 9305837

[B42] Luger K. Dechassa M. L. Tremethick D. J. (2012). New insights into nucleosome and chromatin structure: an ordered state or a disordered affair? Nat. Rev. Mol. Cell Biol. 13, 436–447. 10.1038/nrm3382 22722606 PMC3408961

[B43] Ma Y. Kanakousaki K. Buttitta L. (2015). How the cell cycle impacts chromatin architecture and influences cell fate. Front. Genet. 6, 19. 10.3389/fgene.2015.00019 25691891 PMC4315090

[B44] Magalska A. Schellhaus A. K. Moreno-Andres D. Zanini F. Schooley A. Sachdev R. (2014). RuvB-like ATPases function in chromatin decondensation at the end of mitosis. Dev. Cell 31, 305–318. 10.1016/j.devcel.2014.09.001 25443297

[B45] Mechali M. Yoshida K. Coulombe P. Pasero P. (2013). Genetic and epigenetic determinants of DNA replication origins, position and activation. Curr. Opin. Genet. Dev. 23, 124–131. 10.1016/j.gde.2013.02.010 23541525

[B46] Mirny L. A. Imakaev M. Abdennur N. (2019). Two major mechanisms of chromosome organization. Curr. Opin. Cell Biol. 58, 142–152. 10.1016/j.ceb.2019.05.001 31228682 PMC6800258

[B47] Nagano T. Lubling Y. Varnai C. Dudley C. Leung W. Baran Y. (2017). Cell-cycle dynamics of chromosomal organization at single-cell resolution. Nature 547, 61–67. 10.1038/nature23001 28682332 PMC5567812

[B48] Nair N. Shoaib M. Sorensen C. S. (2017). Chromatin dynamics in genome stability: roles in suppressing endogenous DNA damage and facilitating DNA repair. Int. J. Mol. Sci. 18 (7), 1486. 10.3390/ijms18071486 PMC5535976 28698521

[B49] Naumova N. Imakaev M. Fudenberg G. Zhan Y. Lajoie B. R. Mirny L. A. (2013). Organization of the mitotic chromosome. Science 342, 948–953. 10.1126/science.1236083 24200812 PMC4040465

[B50] Nemeth A. Conesa A. Santoyo-Lopez J. Medina I. Montaner D. Peterfia B. (2010). Initial genomics of the human nucleolus. PLoS Genet. 6, e1000889. 10.1371/journal.pgen.1000889 20361057 PMC2845662

[B51] Nishioka K. Rice J. C. Sarma K. Erdjument-Bromage H. Werner J. Wang Y. (2002). PR-Set7 is a nucleosome-specific methyltransferase that modifies lysine 20 of histone H4 and is associated with silent chromatin. Mol. Cell 9, 1201–1213. 10.1016/S1097-2765(02)00548-8 12086618

[B52] Nozaki T. Imai R. Tanbo M. Nagashima R. Tamura S. Tani T. (2017). Dynamic organization of chromatin domains revealed by super-resolution live-cell imaging. Mol. Cell 67 282–293, e287. 10.1016/j.molcel.2017.06.018 28712725

[B53] Nunez E. Fu X. D. Rosenfeld M. G. (2009). Nuclear organization in the 3D space of the nucleus - cause or consequence? Curr. Opin. Genet. Dev. 19, 424–436. 10.1016/j.gde.2009.07.005 19846290 PMC2796509

[B54] Oda H. Okamoto I. Murphy N. Chu J. Price S. M. Shen M. M. (2009). Monomethylation of histone H4-lysine 20 is involved in chromosome structure and stability and is essential for mouse development. Mol. Cell Biol. 29, 2278–2295. 10.1128/MCB.01768-08 19223465 PMC2663305

[B55] Palozola K. C. Donahue G. Liu H. Grant G. R. Becker J. S. Cote A. (2017). Mitotic transcription and waves of gene reactivation during mitotic exit. Science 358, 119–122. 10.1126/science.aal4671 28912132 PMC5727891

[B56] Piskadlo E. Oliveira R. A. (2017). A topology-centric view on mitotic chromosome architecture. Int. J. Mol. Sci. 18 (12), E2751. 10.3390/ijms18122751 29258269 PMC5751350

[B57] Poleshko A. Smith C. L. Nguyen S. C. Sivaramakrishnan P. Wong K. G. Murray J. I. (2019). H3K9me2 orchestrates inheritance of spatial positioning of peripheral heterochromatin through mitosis. Elife 8, e49278. 10.7554/eLife.49278. 31573510 PMC6795522

[B58] Pope B. D. Ryba T. Dileep V. Yue F. Wu W. Denas O. (2014). Topologically associating domains are stable units of replication-timing regulation. Nature 515, 402–405. 10.1038/nature13986 25409831 PMC4251741

[B59] Potapova T. A. Gerton J. L. (2019). Ribosomal DNA and the nucleolus in the context of genome organization. Chromosome Res. 27, 109–127. 10.1007/s10577-018-9600-5 30656516

[B60] Qian J. Lesage B. Beullens M. Van Eynde A. Bollen M. (2011). PP1/Repo-man dephosphorylates mitotic histone H3 at T3 and regulates chromosomal aurora B targeting. Curr. Biol. 21, 766–773. 10.1016/j.cub.2011.03.047 21514157

[B61] Raccaud M. Suter D. M. (2018). Transcription factor retention on mitotic chromosomes: regulatory mechanisms and impact on cell fate decisions. FEBS Lett. 592, 878–887. 10.1002/1873-3468.12828 28862742

[B62] Ragoczy T. Telling A. Scalzo D. Kooperberg C. Groudine M. (2014). Functional redundancy in the nuclear compartmentalization of the late-replicating genome. Nucleus 5, 626–635. 10.4161/19491034.2014.990863 25493640 PMC4615584

[B63] Ramadan K. Bruderer R. Spiga F. M. Popp O. Baur T. Gotta M. (2007). Cdc48/p97 promotes reformation of the nucleus by extracting the kinase Aurora B from chromatin. Nature 450, 1258–1262. 10.1038/nature06388 18097415

[B64] Rao S. S. Huntley M. H. Durand N. C. Stamenova E. K. Bochkov I. D. Robinson J. T. (2014). A 3D map of the human genome at kilobase resolution reveals principles of chromatin looping. Cell 159, 1665–1680. 10.1016/j.cell.2014.11.021 25497547 PMC5635824

[B65] Remus D. Diffley J. F. (2009). Eukaryotic DNA replication control: lock and load, then fire. Curr. Opin. Cell Biol. 21, 771–777. 10.1016/j.ceb.2009.08.002 19767190

[B66] Sati S. Cavalli G. (2017). Chromosome conformation capture technologies and their impact in understanding genome function. Chromosoma 126, 33–44. 10.1007/s00412-016-0593-6 27130552

[B67] Sawicka A. Seiser C. (2012). Histone H3 phosphorylation - a versatile chromatin modification for different occasions. Biochimie 94, 2193–2201. 10.1016/j.biochi.2012.04.018 22564826 PMC3480636

[B68] Schalbetter S. A. Goloborodko A. Fudenberg G. Belton J. M. Miles C. Yu M. (2017). SMC complexes differentially compact mitotic chromosomes according to genomic context. Nat. Cell Biol. 19, 1071–1080. 10.1038/ncb3594 28825700 PMC5640152

[B69] Shoaib M. Walter D. Gillespie P. J. Izard F. Fahrenkrog B. Lleres D. (2018). Histone H4K20 methylation mediated chromatin compaction threshold ensures genome integrity by limiting DNA replication licensing. Nat. Commun. 9, 3704. 10.1038/s41467-018-06066-8 30209253 PMC6135857

[B70] Simonis M. Klous P. Splinter E. Moshkin Y. Willemsen R. De Wit E. (2006). Nuclear organization of active and inactive chromatin domains uncovered by chromosome conformation capture-on-chip (4C). Nat. Genet. 38, 1348–1354. 10.1038/ng1896 17033623

[B71] Sivakumar A. De Las Heras J. I. Schirmer E. C. (2019). Spatial genome organization: from development to disease. Front. Cell Dev. Biol. 7, 18. 10.3389/fcell.2019.00018 30949476 PMC6437099

[B72] Tardat M. Brustel J. Kirsh O. Lefevbre C. Callanan M. Sardet C. (2010). The histone H4 Lys 20 methyltransferase PR-Set7 regulates replication origins in mammalian cells. Nat. Cell Biol. 12, 1086–1093. 10.1038/ncb2113 20953199

[B73] Tremethick D. J. (2007). Higher-order structures of chromatin: the elusive 30 nm fiber. Cell 128, 651–654. 10.1016/j.cell.2007.02.008 17320503

[B74] Vagnarelli P. Ribeiro S. Sennels L. Sanchez-Pulido L. De Lima Alves F. Verheyen T. (2011). Repo-Man coordinates chromosomal reorganization with nuclear envelope reassembly during mitotic exit. Dev. Cell 21, 328–342. 10.1016/j.devcel.2011.06.020 21820363 PMC3480639

[B75] Van Koningsbruggen S. Gierlinski M. Schofield P. Martin D. Barton G. J. Ariyurek Y. (2010). High-resolution whole-genome sequencing reveals that specific chromatin domains from most human chromosomes associate with nucleoli. Mol. Biol. Cell 21, 3735–3748. 10.1091/mbc.e10-06-0508 20826608 PMC2965689

[B76] Van Nuland R. Gozani O. (2016). Histone H4 lysine 20 (H4K20) methylation, expanding the signaling potential of the proteome one methyl moiety at a time. Mol. Cell Proteomics 15, 755–764. 10.1074/mcp.R115.054742 26598646 PMC4813698

[B77] Van Steensel B. Belmont A. S. (2017). Lamina-associated domains: links with chromosome architecture, heterochromatin, and gene repression. Cell 169, 780–791. 10.1016/j.cell.2017.04.022 28525751 PMC5532494

[B78] Vankova Hausnerova V. Lanctot C. (2017a). Chromatin decondensation is accompanied by a transient increase in transcriptional output. Biol. Cell 109, 65–79. 10.1111/boc.201600032 27633335

[B79] Vankova Hausnerova V. Lanctot C. (2017b). Transcriptional output transiently spikes upon mitotic exit. Sci. Rep. 7, 12607. 10.1038/s41598-017-12723-7 28974707 PMC5626720

[B80] Vermeulen M. Eberl H. C. Matarese F. Marks H. Denissov S. Butter F. (2010). Quantitative interaction proteomics and genome-wide profiling of epigenetic histone marks and their readers. Cell 142, 967–980. 10.1016/j.cell.2010.08.020 20850016

[B81] Vertii A. Ou J. Yu J. Yan A. Pages H. Liu H. (2019). Two contrasting classes of nucleolus-associated domains in mouse fibroblast heterochromatin. Genome Res. 29, 1235–1249. 10.1101/gr.247072.118 31201210 PMC6673712

[B82] Wang F. Higgins J. M. (2013). Histone modifications and mitosis: countermarks, landmarks, and bookmarks. Trends Cell Biol. 23, 175–184. 10.1016/j.tcb.2012.11.005 23246430

[B83] Wood A. J. Severson A. F. Meyer B. J. (2010). Condensin and cohesin complexity: the expanding repertoire of functions. Nat. Rev. Genet. 11, 391–404. 10.1038/nrg2794 20442714 PMC3491780

[B84] Yeeles J. T. Deegan T. D. Janska A. Early A. Diffley J. F. (2015). Regulated eukaryotic DNA replication origin firing with purified proteins. Nature 519, 431–435. 10.1038/nature14285 25739503 PMC4874468

[B85] Zaidi S. K. Nickerson J. A. Imbalzano A. N. Lian J. B. Stein J. L. Stein G. S. (2018). Mitotic gene bookmarking: an epigenetic program to maintain normal and cancer phenotypes. Mol. Cancer Res. 16, 1617–1624. 10.1158/1541-7786.MCR-18-0415 30002192 PMC6214712

[B86] Zhang H. Emerson D. J. Gilgenast T. G. Titus K. R. Lan Y. Huang P. (2019). Chromatin structure dynamics during the mitosis-to-G1 phase transition. Nature 576, 158–162. 10.1038/s41586-019-1778-y 31776509 PMC6895436

